# Changes in the Dissolved Organic Matter Characteristics Released from Sediment According to Precipitation in the Namhan River with Weirs: A Laboratory Experiment

**DOI:** 10.3390/ijerph19094958

**Published:** 2022-04-19

**Authors:** Haeseong Oh, Jung-Hyun Choi

**Affiliations:** Department of Environmental Science and Engineering, Ewha Womans University, 52, Ewhayeodae-gil, Seodaemun-gu, Seoul 03760, Korea; hs1226@ewhain.net

**Keywords:** dissolved organic matter (DOM), release rate, sediment, sediment-water interface, optical properties

## Abstract

In this study, changes in the properties of dissolved organic matter (DOM) released from sediments into water layers were investigated. To analyze the spatial and temporal variation in dissolved organic carbon (DOC), sediment and bottom water samples were collected upstream of the Gangcheon, Yeoju, and Ipo weirs of the Namhan River during the rainy and non-rainy seasons. The initial DOC was correlated with precipitation (R^2^ = 0.295, *p* = 0.034) and residence time (R^2^ = 0.275, *p* = 0.040). The change in the bottom water DOC concentration resulted from the DOC released from the sediments, which may cause water quality issues in the bottom water. The fluorescence analysis revealed that the DOM contained higher levels of hydrophilic and low-molecular-weight (LMW) organic matter in the non-rainy season and higher levels of hydrophobic and high-molecular-weight (HMW) organic matter in the rainy season. Since the Namhan River is the main resource of drinking water for the Seoul metropolitan area, our results can help to optimize the drinking water treatment process by reflecting the DOM characteristics that vary with the seasons. Furthermore, the statistical analysis confirmed that the nutrient content of pore-water and sediment can be used to estimate the DOM release rate from the sediment to the water layer. The results of this study provide a better understanding of DOM movement in aquatic ecosystems and the influences of rainfall on the water quality of the surface waterbody.

## 1. Introduction

DOM is an important source of carbon for rivers [1,2]. As the main energy source for microbial activity, DOM can affect ecological and biogeochemical processes in rivers [3,4,5]. The role of DOM in biogeochemical and ecological processes depends on its source, composition, the physicochemical conditions of the river, and the biological community [6,7]. DOM is classified into autochthonous and allochthonous depending on the source and composition. Autochthonous DOM is produced in the water body by phytoplankton, zooplankton, and a mixture of living and dead bacteria [8,9]. It mainly consists of non-humic substances that are labile and easily degraded by microorganisms [4,10]. Allochthonous DOM originates from leaves, eroded soils from the terrain, and organic wastes of anthropogenic origin, such as organic fertilizers, wastewaters, and industrial effluents [11]. Allochthonous DOM usually consists of humic substances with high molecular weight and lignin content, and it has the possibility to accumulate in sediments more than autochthonous components [9,10]. 

The quantity and quality of sediment DOM can be affected by anthropogenic activities. The watershed characteristics and the hydrologic alteration resulting from the construction of weirs have impacts on the sedimentation rates and the quantity and quality of DOM buried in the sediment. Since the sediment’s DOM can be released into the overlying water through resuspension, erosion, and disturbance [12], the river with weir-impoundment may potentially raise water quality issues arising from the continuous release of DOM from sediments. Most especially, since the increase in DOM has been closely associated with disinfection byproduct (DBP) formation potentials [13], it is essential to obtain additional insight into the dynamics of sediment DOM in such a weir-impounded river. The concentration and composition of DOM that is released from the sediment can vary depending on the environmental factors of the sediment and water layer, such as particle size; the temperature and DO concentration of the water; the nutrient content of the sediment, pore-water, and water; and hydrological characteristics [14,15]. As DOM movement from sediments to the water layer results from a combination of various mechanisms, it is difficult to identify the environmental factors that predominantly contribute to this process [16].

In particular, rainfall, among other environmental factors, has the potential to affect the DOM of the river and DOM release from the sediment. Organic matter contained in upstream watersheds and surrounding terrestrial environments can be transported into rivers through runoff by rainfall. The allochthonous DOM contained in runoff flow into rivers and can change the DOM concentration and properties of the river [11,17,18]. Previous studies have demonstrated that river DOM concentrations are associated with the rainfall–runoff processes during the rainy season [19,20,21]. In addition, the intensity and frequency of rainfall have been associated with the sediment accumulation and the quantity and quality of DOM buried in the sediment. If the flow velocity of the sediment–water interface is increased by the rainfall–runoff processes, DOM can be released from the river sediment to the water layer [19,20]. Therefore, the nutrient dynamics of sediment in rivers are related to rainfall–runoff processes and the intensity and frequency of rainfall [11]. 

The measurements of optical properties, such as absorption and fluorescence spectroscopy, have been widely utilized to investigate the composition, distribution, and dynamics of DOM in aquatic systems over recent decades [19,22,23,24,25]. In particular, fluorescence spectroscopy has been used to characterize DOM with high sensitivity, simplicity, and reliability [14,23,26]. Specific ultraviolet absorbance (SUVA_254_), which is used to monitor DOM optical properties, can be used to estimate the presence of aromatic organic carbon compounds in DOM according to rainfall. SUVA_254_ is high when HMW organic matter originating from the humus in forests and soils is introduced into rivers through rainfall [27]. An increase in river water level owing to rainfall can enhance the release rate of soluble organic carbon from the sediments as well as the DOC concentrations in the bottom water layer [11,28,29]. Determining the release rate and the chemical composition of DOM is crucial for understanding both the fate of the sediment DOM and the ecological and biogeochemical effects of DOM in the bottom water.

In an aquatic ecosystem, sediment serves as a source of DOM through release to the bottom water. Considering the accumulation and the release of the sediment affected by rainfall, it would be highly valuable to explore the quantity and quality of DOM released from the sediment depending on the effect of the rainfall. Most previous studies have primarily applied optical techniques to characterize DOM in the river water after rainfall. However, little research has been conducted to investigate the DOM released from the sediment after rainfall [15,30,31]. Therefore, this study investigated the DOM released from sediment samples with spatial and seasonal variations using incubation experiments. The objectives of our study are to: (1) understand the contribution of the DOM released from sediment to the quantity and quality of the overlying water via incubation, (2) analyze the optical properties of the DOM released from sediment using fluorescence analysis, and (3) identify the essential environmental factors that affect the DOC release rate and optical properties of DOM via statistical analyses. The results of this study will have implications for better restraining DOM movement in an aquatic ecosystem and for inferring the influences of environmental factors, especially rainfall, on the quantity and quality of DOM in bottom water.

## 2. Materials and Methods

### 2.1. Site Description

This study was conducted within the Namhan River watershed, which is 375 km long, covers an area of 23,859 km^2^ along the primary river [32,33], and is located in the eastern region of South Korea. There are three artificial weirs in the Namhangang River (Gangcheon weir, Yeoju weir, and Ipo weir). The three weirs are located in Yeoju-si, Gyeonggi-do, Korea. The length, height, and watershed area of the Gangcheon weir are 440 m, 8 m, and 10,972 km^2^. The length, height, and watershed area of Gangcheon Yeoju weir are 513 m, 8 m, and 11,115 km^2^. The length, height, and watershed area of the Ipo weir are 521 m, 6 m, and 11,803 km^2^ [34]. The annual average temperature of the watershed was 13.0 °C (−13.5–30.0 °C) in 2016 and 12.3 °C (−9.8–29.9 °C) in 2017. The East Asian summer monsoon climate influences the hydrologic characteristics of the watershed, with over 50% of the annual precipitation falling in July and August. The annual precipitation was 935.9 mm from 89 days of rain or snow at a rate of over 0.1 mm/day in 2016 and 1188.4 mm from 111 days in 2017 [35].

### 2.2. Sampling and Sample Preparation

**Figure 1:**
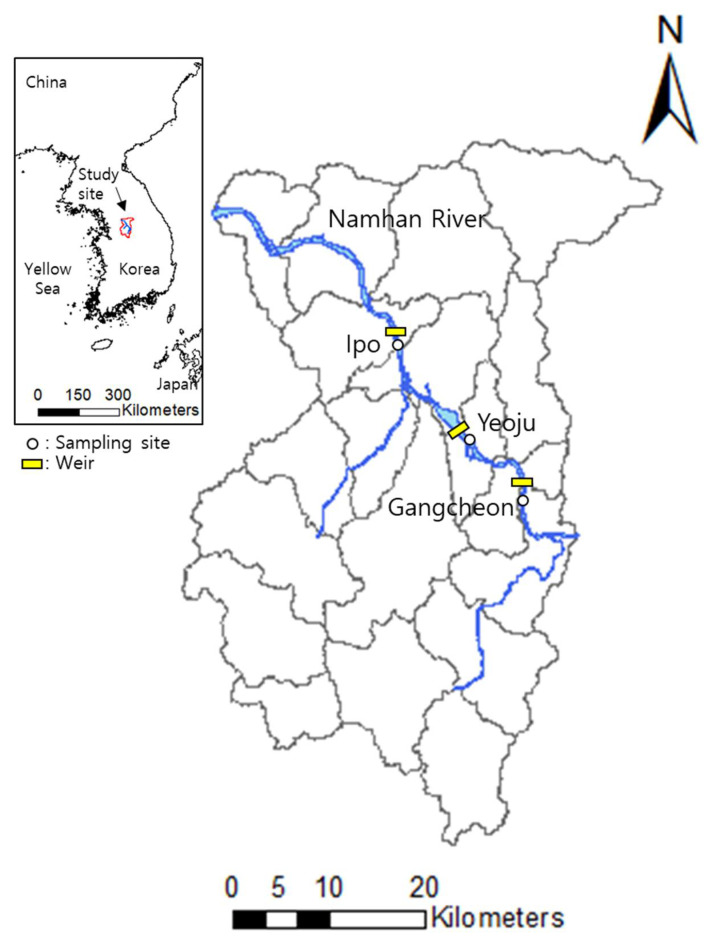
Map of sampling locations in the Namhan River basin, Korea. Detailed sampling site indications are provided (Gangcheon, Yeoju, and Ipo weir).

As part of a large river restoration project designed to control flooding and water supply, multipurpose weirs have been constructed along the Namhan River (Figure 1). Weir construction is known to exert impacts on the quantity and quality of sediment DOM by facilitating the accumulation of sediments near the weir [18]. On four different days between August 2016 and June 2017, sediment samples were collected upstream of the Gangcheon (37°16′29.50″ N, 127°41′13.70″ E), Yeoju (37°19′9.10″ N, 127°37′14.80″ E), and Ipo weir (37°24′8.30″ N, 127°32′29.00″ E).

For incubation, acrylic core samplers with a rubber stopper (diameter and height of 10 and 22 cm, respectively) were used to collect a 25 cm long sediment core together with the overlying water by a scuba diver. Three sediment cores were sampled for incubation (n = 1), for the analysis of the sediment (n = 1), and pore-water (n = 1) chemistry at each weir. The bottom water was separately collected in separate bottles using a Niskin water sampler. The core and bottom water samples were carefully placed vertically in an ice box while they were transported to the laboratory [36].

**Figure 2:**
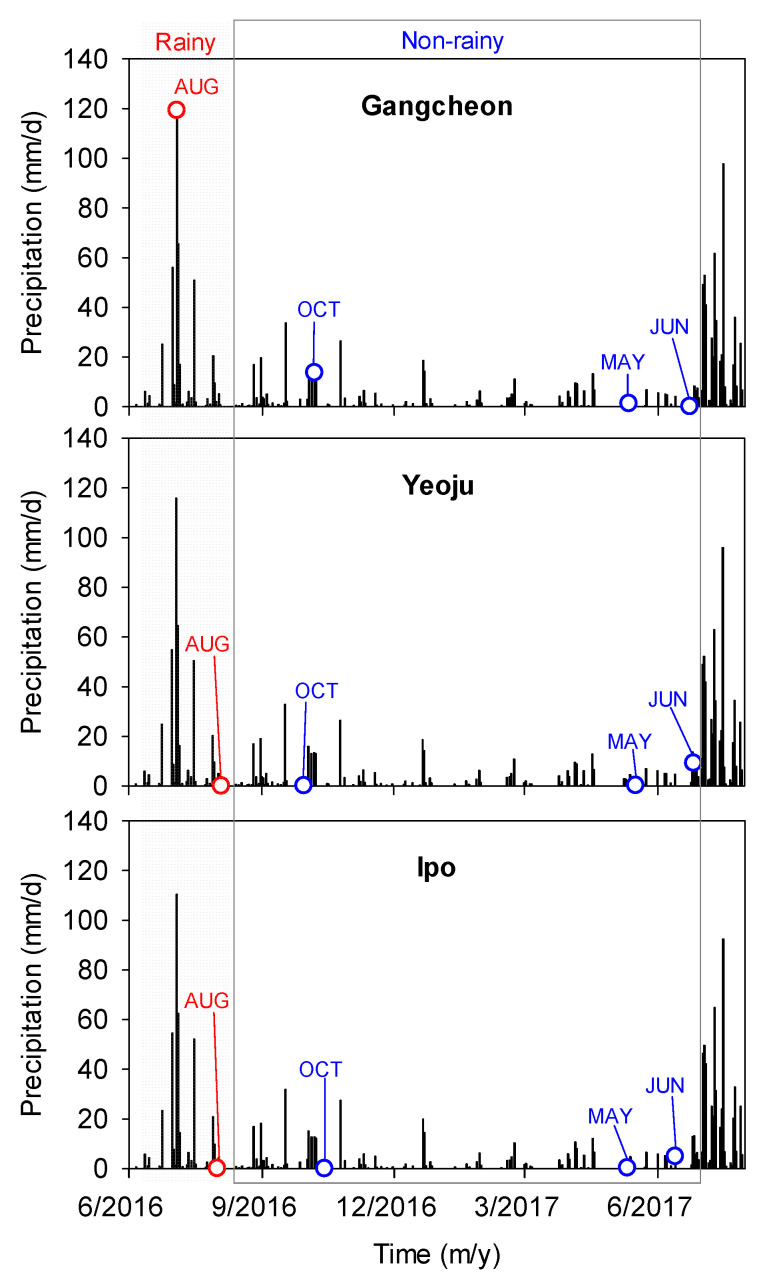
Precipitation during the sampling period.

Given that rainfall has an effect on sediment composition and characteristics, the sampling was divided into two periods: rainy and non-rainy seasons. Based on close observation of the rainfall pattern in the Namhan River watershed, the samples collected in August 2016 were classified as rainy season samples, whereas those collected in October 2016, May 2017, and June 2017 were classified as non-rainy season samples (Figure 2). In particular, water flowing into rivers through the watershed owing to rainfall contains allochthonous origin pollution, especially OM [19]. The DOM optical properties in river sediments depend on the land type and use in the watershed [37].

The sediment samples were retrieved from the upper part (0–2 cm) of the core and homogeneously mixed in a nitrogen-purged vinyl bag, Figure S1) [38]. The homogeneously mixed sediment (45 g) was placed in a 100 mL serum vial containing 70 mL of bottom water filtered using Whatman GF/F filters (0.7 μm) [10]. The filtered bottom water was poured into the vial, being careful not to mix it with the sediment. The filtered river water was used for incubation because the incubation experiment reproduces the real environmental conditions of the study area. The top of the vials was packed with glass wool to prevent the evaporation of the water and the vials were not shaken during incubation. The vials containing sediment and the bottom water were incubated to estimate the change in OM concentration released from the sediments into the water layer. The changes in OM concentration can help understand the contribution of the DOM released from the sediments to the condition of the water layer, such as anoxia and eutrophication.

Laboratory incubation was carried out in a dark chamber where the ambient temperature was kept constant at 20 °C for up to 7 days [39,40]. The average residence time of the weirs constructed in 4 major rivers (Han, Geum, Nakdong, and Youngsan River) in South Korea was 2.46 ± 2.78 days for the rainy season and 7.63 ± 8.25 days for the non-rainy season in 2016–2017. Therefore, the sample vials were incubated for 3 and 7 days. A total of 9 vials were incubated at each site (Gangcheon, Yeoju, and Ipo). At 0, 3, and 7 days of incubation, 3 vials from each site were sacrificed for analysis. In addition, in August and October 2016, vials containing bottom water without sediments (control) were incubated under the same conditions to estimate the change in DOM influenced by the bottom water only.

### 2.3. Analytical Methods

After incubation, the supernatant collected without disturbing the sediment in the vials was filtered [39], and DOC concentrations were measured using a total organic carbon (TOC) analyzer (V-CPH, Shimadzu, Kyoto, Japan). After removing the particulate organic carbon using a GF/F filter, the DOC was measured using a non-purgeable organic carbon (NPOC) method. The DOC release rate (mg/m^2^/day) was calculated as the difference in the concentration of DOC between 0 and 3 Days (DOC_3day_–DOC_0day_), multiplied by the volume of the supernatant in the sample vial and divided by the area of the surface sediment and the number of days of incubation [41].

The absorbance spectra from 100 to 900 nm were measured using a UV-visible spectrophotometer (Libra S32 PC, Biochrom, Cambridge, UK). Fluorescence intensity was determined using the fluorescence excitation–emission matrix (EEM) technique (F-7000, Hitachi, Tokyo, Japan). The EEMs were scanned at excitation wavelengths of 200–400 nm with a stepwise increase of 5 nm and at emission wavelengths of 290–540 nm with 1 nm increments. Excitation and emission slits were both adjusted to 5 nm.

The absorbance and fluorescence intensities of the samples were used to calculate SUVA_254_, the humification index (HIX), and the fluorescence index (FI). SUVA_254_, which is an indicator of the presence of aromatic organic carbon compounds in DOM, is calculated by dividing the absorbance at 254 nm by the DOC concentration [42]. If it is higher than 4, it implies that the DOM predominantly consists of hydrophobic and HMW organic matter. If it is less than 2, it implies that hydrophilic and LMW organic matter are dominant [43]. 

The HIX was calculated using the 435–480 nm to 300–435 nm ratio of the spectral region areas of the emission spectra at an excitation wavelength of 254 nm [44]. The HIX, which is often considered an indicator of DOM maturity, represents the degree of humification [44]. It increases as microorganism-induced humification increases [23], i.e., if it is below 10, the DOM is not strongly humified and contains more oxygen-containing functional groups [44].

The FI, which is an indicator of OM origin, was calculated using the ratios of the emission intensity at 450 nm and 500 nm to excitation at 370 nm [25]. FI is an indicator of OM origin; when it is below 1.4, it implies the presence of significant levels of terrestrial and aromatic organic carbon; when it is above 1.9, it implies the presence of organic material with low amounts of aromatic organic carbon originating from microorganisms [25].

PARAFAC analysis was performed on the 107 EEMs generated from the incubated samples collected from the Gangcheon, Yeoju, and Ipo weirs. Using two components, the model generated the best statistical results and was able to explain more than 99.9% of the EEMs. PARAFAC modeling was conducted using MATLAB 7.6 (MathWorks, Natick, MA, USA) with the N-way toolbox [45]. A total of 107 EEMs from Gangcheon, Yeoju, and Ipo for the four sampling periods were identified and used to produce two representative PARAFAC components.

The in situ temperature and DO concentration for the surface and bottom water layers were measured using a multiparameter instrument (professional plus, YSI, Yellow Springs, OH, USA). The physical and chemical characteristics of the sediments (0–2 cm), including particle size, pore-water nitrogen (total dissolved nitrogen (TDN) and dissolved organic nitrogen (DON)), and sediment TOC, were measured. 

Particle size was determined by sieving the sediments and using an automatic particle size analyzer after removing carbonate and OM using hydrogen peroxide (Mastersizer 2000, Mastersizer, Malvern, UK). Samples for the pore-water DON and TDN concentrations were collected using a Rhizon sampler (Rhizon CCS, Rhizosphere Research Products, Wageningen, The Netherland). The TDN in the pore-water samples was measured by a total organic carbon analyzer (TOC-VCPN, Shimadzu, Kyoto, Japan). The DON concentration was calculated by subtracting the dissolved inorganic nitrogen (NH_3_-N, NO_2_-N, and NO_3_-N) from the TDN. Concentrations of NH_3_-N were determined based on the phenate method (standard methods 4500-NH3), and the concentrations of NO_2_-N and NO_3_-N were measured by ion chromatography (790 Personal IC, Metrohm, Herisau, Switzerland). The sediment samples were freeze-dried and crushed in an agate mortar. An elemental analyzer (EA 1110, GV Instruments, Manchester, UK) was used to determine the TOC after removing inorganic carbonate using 1.0 M hydrochloric acid.

In the rainy season, a large amount of contaminants flows into rivers with surface runoff by rainfall [46,47]. In addition, the inflow of surface runoff increases the flow rate of a river, which affects the transport and accumulation of sediment [48]. Therefore, the residence time and precipitation of weirs are considered environmental factors that affect the quantity and quality of sediment. The residence time was calculated by dividing the volume of the water body by the inflowing water volume based on data provided by the Korea Water Resources Corporation [34]. For precipitation, the previous 1-month average precipitation was provided by K-water, which was used depending on the sampling location and date.

### 2.4. Statistical Analyses

To investigate significant differences between the sampling sites and periods with respect to the physical and chemical characteristics of the samples, such as particle size, surface and bottom water DO concentrations and temperature, pore-water TDN, DON, sediment TOC, residence time, and precipitation, one-way analysis of variance (ANOVA) with Tukey’s test (equal variance) or Dunnett’s T3 test (unequal variance) for post-hoc analysis was conducted (IBM SPSS Statistics, IBM, New York, NY, USA) [49]. A linear regression analysis of the relationship between the DOM properties and environmental factors was performed (Sigmaplot 10.0, Systat Software, San Jose, CA, USA). A principal component analysis (PCA) was also conducted to illustrate the association of DOC and the optical properties of DOM and environmental factors using Unscrambler X 10.1 (CAMO Software, Oslo, Norway) [50].

## 3. Results and Discussion

### 3.1. Physical and Chemical Characteristics of the Study Area

The sediment particle size, temperature, and DO concentration of the surface and bottom water layers were measured at the sampling sites, and the results are shown in Table 1. The average surface and bottom water temperatures were 24.40 ± 2.17 °C and 25.10 ± 2.85 °C in the rainy season (August 2016) and 20.61 ± 2.878 °C and 20.21 ± 2.86 °C in the non-rainy season (Table 1). The temperatures of the surface and bottom water layers were significantly higher in the rainy season (August 2016) than in the non-rainy season (May 2017; *p* < 0.05). Additionally, the differences between the surface and bottom water temperatures were below 2 °C across all sampling sites and sampling periods, and no stratification was observed. The DO concentrations of the surface and bottom water layers were 8.91 ± 1.21 mg/L and 8.52 ± 1.555 mg/L, respectively. Since the degree of DO saturation of the bottom water layer was in the range of 71–135%, no oxygen depletion was observed. The sediment particle size ranged from 0.013 mm to 0.363 mm at the Gangcheon weir, from 0.029 mm to 0.068 mm at the Yeoju weir, and from 0.056 mm to 0.230 mm at the Ipo weir. There were no significant differences between the sampling sites and sampling periods with respect to particle size and DO concentrations (*p* > 0.05). Pore-water TDN and DON concentrations were in the ranges of 1.758–8.785 mg/L and 0.607–5.118 mg/L, respectively. There were no significant differences in sampling periods and sites (*p* > 0.05). The previous study conducted on the sediments of four major rivers showed that the concentration of TDN was 12.08 ± 8.90 mg/L in July–August and 9.63 ± 6.36 mg/L in September–October of 2016 [36]. The DON concentration was 12.00 ± 8.93 mg/L in July–August and 9.34 ± 6.42 mg/L in September–October in the same study [36]. From a comparison of the concentrations of TDN (4.460 mg/L in August and 4.027 ± 2.126 mg/L in October) and DON (4.320 mg/L in August and 3.260 ± 2.357 mg/L in October) in this study, it can be concluded that the Namhan River has lower concentrations of pore-water TDN and DON than other rivers in Korea (including the Geum, Yeongsan, and Nakdong Rivers). The TOC concentration of the surface sediment was in the range of 0.36–3.59%, and also had no statistical difference between temporal and spatial variables. The residence time was 0.575 ± 0.301 d in the rainy season and 1.906 ± 0.396 d in the non-rainy season. The monthly average precipitation was 7.190 ± 3.209 mm/d in the rainy season and 1.515 ± 1.012 mm/d in the non-rainy season. There was a significant difference in the residence time and precipitation between the sampling periods, the rainy and non-rainy seasons (*p* < 0.05).

### 3.2. Dissolved Organic Carbon (DOC)

**Figure 3:**
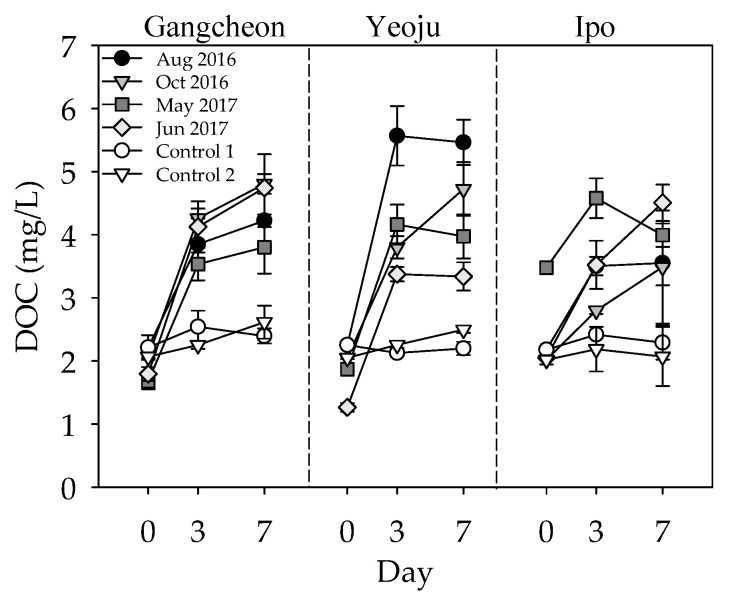
Changes in the DOC concentration in the samples and two controls during the incubation period.

The initial DOC concentration (Day 0) in the bottom water from the Gangcheon, Yeoju, and Ipo weirs was 1.27–3.48 mg/L (Figure 3). There were significant differences in the initial concentration of DOC in the rainy season (average 2.21 ± 0.04 mg/L) and non-rainy season (average 2.03 ± 0.60 mg/L; *p* < 0.05). However, there were no significant differences in the initial DOC among the sampling sites. The DOC concentration in the water layer tended to increase according to the incubation time, and the rate of increase within the first three days of incubation was higher than that observed within days 3–7. The DOC release rate between 0 and 3 days of incubation was in the range of 0.015–0.062 mg/m^2^/day. There were no significant differences in the DOC release rate in the rainy season (0.025–0.062 mg/m^2^/day) and non-rainy season (0.015–0.043 mg/m^2^/day, *p* > 0.05). Measuring the DOC release rate with respect to each weir, for the Yeoju weir, the DOC release rates in the rainy season were significantly higher than those in the non-rainy season (*p* < 0.05). The DOC release rates of other weirs in the rainy season were higher than the average value of the non-rainy season. In addition, the DOC release rate in the weirs located upstream in the Namhan River, such as Gangcheon and Yeoju, were higher than the Ipo weir (*p* < 0.05).

To examine the effect of sediment on DOC concentration, the bottom water without the sediments (control) was incubated under the same conditions as the sample vials containing the sediments (Figure 3). The DOC concentration increased from −4 to 22% (average, 11%) in the control and from 78 to 104% (average, 89%) in the sample with the sediment. The results of the control indicated that the increase observed in the sample vials containing the sediments primarily resulted from the DOC released from the sediments. During the incubation, the apparent DOC increase in the sample vials containing the sediment may result not only from physical transport such as diffusion but also from various biogeochemical processes such as microbial degradation at the sediment-water interface. From the result of incubation, it could be concluded that DOC released from sediment plays an essential role in increasing bottom-water DOC concentration.

**Figure 4:**
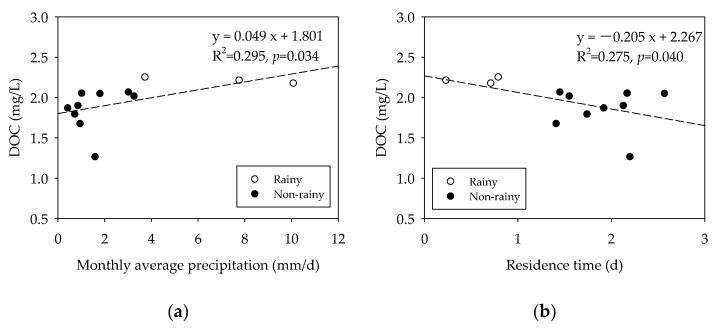
Correlation between (**a**) the initial DOC concentration (day 0) and monthly average precipitation; (**b**) the initial DOC concentration (day 0) and the residence time.

Since the initial DOC concentration was different depending on the sampling period, the relationship between DOC concentration and the sampling period was examined. The main characteristics, the amount of precipitation and residence time, were selected to represent the sampling period of the rainy and non-rainy seasons. The initial DOC concentration was positively correlated with the amount of precipitation (R^2^ = 0.295, *p* = 0.034; Figure 4a) and negatively correlated with residence time (R^2^ = 0.275, *p* = 0.040; Figure 4b). Heavy rainfall results in an increase in river inflow, which leads to a decrease in residence time. Additionally, water flowing from a watershed contains allochthonous pollutants, particularly OM, which has characteristics that depend on the land type and the use of the watershed [21,37]. Based on the relationship between the initial DOC concentration, the amount of precipitation, and the residence time, it can be concluded that environmental factors, such as precipitation and residence time, have an effect on the DOC concentration of the bottom water [19].

### 3.3. Optical Properties of DOM

**Figure 5:**
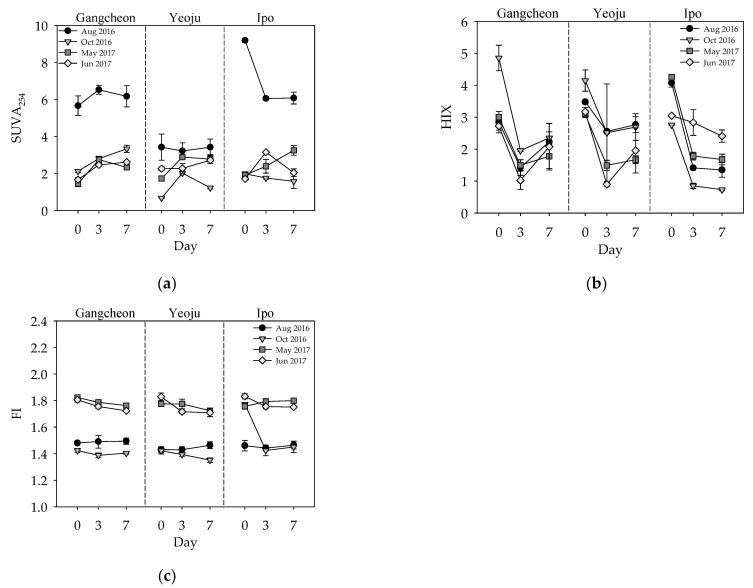
Changes in the optical properties of DOM: (**a**) SUVA_254_, (**b**) HIX, and (**c**) FI during the incubation period.

The initial SUVA_254_ values at the Gangcheon, Yeoju, and Ipo weirs in the non-rainy season were in the range of 0.68–2.26 (Figure 5a), these values were below 4, indicating the predominance of hydrophilic and LMW organic matter. However, it ranged from 3.42 to 9.19 in the rainy season, especially in the Gangcheon and Ipo weirs, the values for which were above 4, indicating the predominance of hydrophobic and HMW organic matter. There were significant differences between rainy and non-rainy seasons with respect to the initial SUVA_254_ (*p* < 0.05). The SUVA_254_ increased during the incubation period, except in August 2016 for the Yeoju weir and August and October for the Ipo weir. The change of SUVA_254_ during the sediment incubation period means that the properties of DOM can be affected by the DOM released from the sediment into the water layer. The SUVA_254_ release rate was significantly different between the rainy season (August 2016) and during the non-rainy season (*p* < 0.05). This could be attributed to the introduction of HMW organic matter into rivers from watersheds during the rainy season [27]. According to previous studies, the SUVA_254_ value has a significant correlation with the DBPs produced in drinking water treatment processes [51,52]. The water layer containing hydrophobic and HMW organic matters in the rainy season, where SUVA_254_ is relatively high, can cause DBPs in water treatment plants on the Namhan River. Therefore, special caution is needed for the operation of drinking water processes during the rainy season compared to the non-rainy season. Regarding the initial SUVA_254_ and SUVA_254_ flux, there were no significant differences between the sampling sites.

The HIX values of the samples collected at the Gangcheon weir were in the range of 2.72–4.85, whereas those of the samples collected at the Yeoju and Ipo weirs were in the ranges of 3.08–4.15 and 2.76–4.25, respectively (Figure 5b). The HIX values tended to decrease with incubation time, and a considerable decrease was observed within the first three days. Since decreases in the HIX can result from a decrease in microorganism-induced humification, and a HIX below 10 means that the DOM is not strongly humified and contains more oxygen-containing functional groups [25,44], based on these observations, it could be concluded that microorganism-induced humification did not occur during the incubation period and that there was an increase in the concentration of functional groups, particularly oxygen-containing functional groups, according to DOC release. The Supplementary Materials Text S1, presents HIX changes of water-extractable organic matter (WEOM) during the incubation. The Supplementary Materials show that the organic matter released from the sediment to the water layer is not humified by microorganisms in the water layer during the incubation period (Figure S3). There were no significant differences between the sampling sites and sampling periods with respect to the initial HIX and HIX flux.

The FI values of the samples collected at the Gangcheon, Yeoju, and Ipo weirs were in the ranges of 1.43–1.82, 1.42–1.83, and 1.46–1.83, respectively (Figure 5c). When FI values are below 1.4, it implies the presence of significant levels of terrestrial and aromatic organic carbon; when they are above 1.9, it implies the presence of organic material with low amounts of aromatic organic carbon originating from microorganisms [25]. Given that the FI values at the three weirs fell within the 1.4 to 1.9 range, it is implied that the samples contained OM originating from both terrestrial and microbial sources [25]. Additionally, the FI values recorded in 2017 were significantly higher than those recorded in 2016 (*p* < 0.0001), because, in 2016, sampling was performed during the rainy season (August 2016). Hence, more terrestrial OM was present relative to the samples collected in 2017. This is consistent with a previous study, which revealed that during the rainy season (August 2016), more DOM originating from terrestrial sources is present relative to other periods [53]. As an indicator of OM origin, there were slight changes in the FI values during the incubation period (*p* > 0.05; except October 2016). There were no significant differences among the sampling sites with respect to initial FI and FI flux

**Figure 6:**
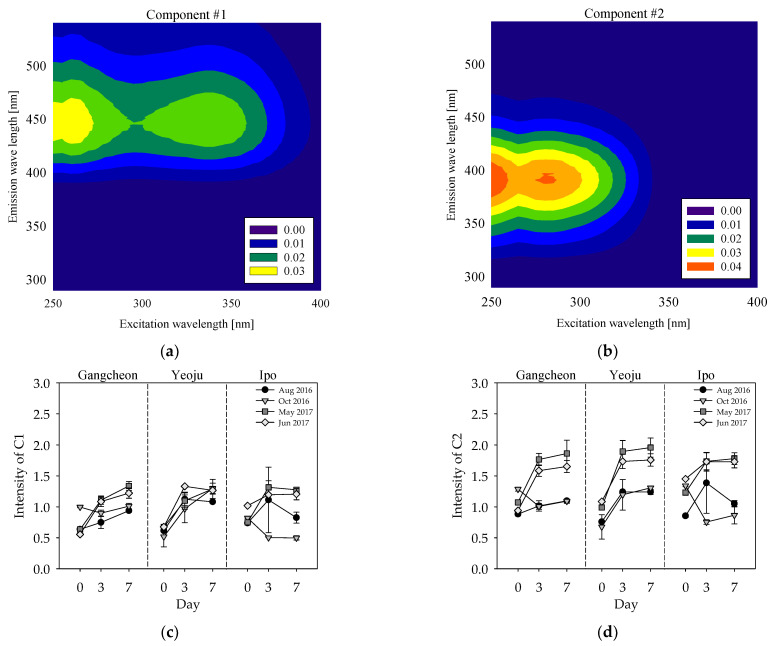
Contour plots of (**a**) Component 1 (terrestrial humic-like components) and (**b**) Component 2 (terrestrial tryptophan-like components; higher intensities in red). Changes in the intensity of PARAFAC components: (**c**) humic-like component (C1) and (**d**) tryptophan-like component (C2).

The results of our PARAFAC analysis using 107 EEMS are shown in Figure 6a,b. The fluorescence components of the incubated samples were estimated from the excitation and emission characteristics of components reported in previous studies. The two fluorescence components are represented as C1 and C2. C1 exhibited maximum peaks at excitations of <250 nm and 330 nm and emissions at 440 nm (Figure 6a). Coble [23] and Stedmon and Markager [54] reported peaks similar to the C1 peaks observed in this study. According to Coble [23], C1 represents humic-like compounds of terrestrial origin found in many natural aquatic systems. C2 showed maximum peaks at excitations of <250 nm and 280 nm and emissions at 370 nm (Figure 6b), which are similar to the peaks reported by Stedmon and Markager [54] and Sanchez et al. [55]. According to these previous studies, C2 resembles protein-like compounds, specifically tryptophan-like substances, originating from autochthonous processes. These tryptophan-like substances are produced by phytoplankton and algae; thus, they likely affect primary productivity in the water layer, resulting in the production of chlorophyll-a.

The change in C1 intensity according to incubation is shown in Figure 6c. The intensity of C1, a terrestrial humic-like substance, generally increased with incubation time. The intensity of the components obtained from PARAFAC indicated the relative concentrations of the corresponding components [56]. Thus, the increase in C1 indicates that the allochthonous origin humic-like substance is contained in the DOC released from the sediment into the water layer. The increase in C1 within the first three days of incubation was greater than that observed within the remaining incubation time. However, the difference in the C1 change between the rainy and non-rainy seasons was not clear.

Similar to the change in C1 intensity, there was an increase in the intensity of C2, a tryptophan-like substance produced by phytoplankton and algae inside the water body (Figure 6d). This means that the DOM containing a tryptophan-like substance was released from the sediment into the water layer. The increase in C2 intensity was statistically greater in the non-rainy season than in the rainy season (*p* < 0.05), when there was no previous precipitation. This result is similar to the FI results for the non-rainy season, where there was more autochthonous origin DOM than allochthonous origin DOM. Therefore, in the non-rainy season without previous rainfall, tryptophan-like substances generated from phytoplankton and algae can affect the DOM concentration of the water layer.

The analysis of the DOM optical properties revealed that the DOM released from sediments to the water layer primarily contained non-humified hydrophilic and LMW organic matter in the non-rainy season. However, during the rainy season in August 2016, hydrophobic and HMW organic matter was released from the sediment. With respect to the origin of the OM, the DOM in the water layer originated from both autochthonous and allochthonous sources. During the rainy season (August 2016), more OM originating from allochthonous sources was observed, and during May–June 2017, the non-rainy season, more OM originating from autochthonous sources, such as tryptophan-like compounds, was observed.

### 3.4. Relationship between DOM and Environmental Factors

Most of the changes in the measured variables were observed within the first three days of incubation; thus, statistical analyses were performed only on data collected during this period. Physical characteristics (surface and bottom temperatures, residence time, precipitation, and sediment particle size) and chemical characteristics (surface and bottom water layer DO, surface sediment TOC concentration, and pore-water TDN and DON contents) were considered environmental factors in this analysis, and all variables were standardized on the same scale.

Linear regression analyses between the properties of DOM (released DOC, SUVA_254_, FI, HIX, C1, and C2) and environmental factors were performed, and the results are shown in Table 2. The correlation between the six DOM properties could be divided into two categories based on their association with environmental factors. The value of Table 2 indicates Pearson’s R-value. If Pearson’s R-value is positive, the properties of DOM and environmental factors have a positive relationship, and if it is negative, it has a negative relationship. The DOC release rate was positively correlated with pore-water TDN, DON, and sediment TOC concentrations (*p* = 0.002, 0.029, and 0.035, respectively). When the pore-water nitrogen concentration and surface sediment organic carbon concentration were high, the concentration of DOC released from the sediment was also high. This finding is consistent with previous studies that DOM release at the sediment–water interface has a major role in transporting and supplying organic carbon and organic nitrogen to microbial populations in the overlying water [57,58]. This result also suggests that sediment carbon is a source of DOC for the water layer [22,59]. Additionally, the HIX release rate, which is an indicator of the degree of humification, was positively correlated with the surface and bottom-layer DO concentrations (*p* = 0.037 and 0.021, respectively). From previous studies, it was known that there is a strong correlation between oxygen concentration and the HIX, since humification occurs more easily in an oxygen-rich environment [60,61]. The results of our research are consistent with those of previous studies.

This is consistent with previous studies, showing that there is a correlation between the HIX and oxygen concentration [62]. From the linear regression results, we confirmed that the DOC release rate, the dissolved nitrogen of the pore-water, and the TOC of the sediment were related and that the HIX was related to the oxygen concentration in the water layer. According to the regression analysis results, sediment-released DOM and its optical properties are influenced by the environmental factors of the bottom water and sediments.

**Figure 7:**
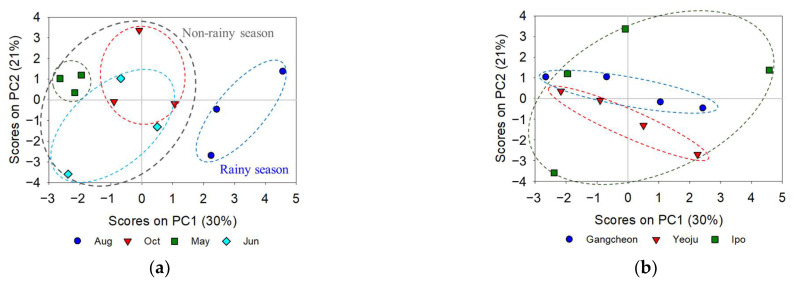
Score plots for PC1 and PC2 as a function of sampling (**a**) time and (**b**) location.

The PCA was performed using data on the DOM optical properties and environmental factors from the Gangcheon, Yeoju, and Ipo weirs collected between August 2016 and June 2017 (four sampling periods). The first two PCs explained more than 50% of the data (PC1: 30% and PC2: 21%) and each point in the scatter plot represents one spectrum. Figure 7a shows that PC1 and PC2 could lead to apparent clustering. The data obtained during the rainy season (August 2016) were located on the positive side of PC1, whereas those obtained during the non-rainy season (October 2016, May and June 2017) tended to be distributed on the negative side of PC1. In addition, precipitation had a strong positive value for PC1 in the loading value, but the residence time had a strong negative value. This means that precipitation and residence time related to the flow rate of the river are highly related to PC1. This result is similar to the ANOVA result in which the initial DOC concentration had a significant correlation with precipitation and residence time. However, there was no obvious difference between the sampling locations (Figure 7b). Thus, the PCA results show that the optical properties of DOM and environmental factors changed with season rather than sampling location.

## 4. Conclusions

In this study, we investigated the spatial and temporal variations in sediment-released DOM and their optical properties to estimate the contribution of the sediment to the quantity and quality of DOM in the overlying water. The results showed that the DOC concentration was a temporal variation with respect to the precipitation and residence time; however, there was no spatial variation. The optical properties of the DOM released from the sediment showed temporal variations. The sediment-released DOM in the rainy season contained higher levels of hydrophobic and HMW organic matter resulting from the allochthonous OM deposited by runoff from the surrounding watershed. In the non-rainy season, the DOM released from the sediment contained more hydrophilic and LMW organic matter resulting from autochthonous sources such as algal activity, microbial activity, and photo-oxidation [18]. Our results showed that the change of bottom-water DOM resulted from the DOM release from the sediments, which may cause water quality issues with respect to the quantity and quality of DOM in the bottom water. Since the Namhan River is the main resource of drinking water for the Seoul metropolitan area, special caution needs to be taken for water treatment during the rainy season when the DOM has a large amount of hydrophobic and HMW organic fractions. Our results can help design and optimize drinking water treatment processes to minimize the risks of DBP production by a timely reflection of the DOM characteristics varying with season and precipitation patterns [13]. The results of this study confirmed that the DOM release rate was significantly correlated with the dissolved nitrogen of pore-water and the organic carbon of sediment. This finding provided a better understanding of the relationship between the DOM released from sediment and environmental factors. However, more research is needed to clarify the spatial and seasonal variability of DOM movement in aquatic ecosystems and to understand its contribution to the quantity and quality of DOM in the surface waterbody.

## Figures and Tables

**Table 1:** Physical and chemical characteristics of the study area.

		Water Depth (m)	Surface Temp. (°C)	Bottom Temp. (°C)	Surface DO (mg/L)	Bottom DO (mg/L)
Gangcheon	range	2.60–5.70	15.90–23.50	15.50–22.00	7.60–10.10	6.30–9.90
	average	3.60 ± 1.46	20.66 ± 3.29	20.05 ± 3.06	8.88 ± 1.26	8.15 ± 1.51
Yeoju	range	1.70–2.20	19.30–25.70	18.50–27.60	7.70–9.80	6.80–10.20
	average	2.03 ± 0.24	22.75 ± 2.87	22.93 ± 3.94	8.68 ± 0.90	8.46 ± 1.63
Ipo	range	1.00–3.50	18.20–25.60	18.10–25.70	7.50–11.40	7.50–11.50
	average	2.25 ± 1.04	21.30 ± 3.71	21.33 ± 3.84	9.20 ± 1.66	8.95 ± 1.87
Rainy season	range	2.20–3.50	21.90–25.70	22.00–27.60	7.50–8.90	7.60–9.50
	average	2.77 ± 0.67	24.40 ± 2.17	25.10 ± 2.85	8.00 ± 0.78	8.57 ± 0.95
Non-rainy Season	range	1.00–5.70	15.90–24.40	15.50–24.40	7.70–11.40	6.30–11.50
	average	2.58 ± 1.35	20.61 ± 2.88	20.21 ± 2.86	9.22 ± 1.19	8.51 ± 1.76

**Table 2:** Pearson correlation (Pearson’s R-value) between DOM properties and environmental factors.

		DOC	SUVA	HIX	FI	C1	C2
Nutrient	Pore-water TDN	0.812 **	0.173	−0.116	0.333	−0.448	−0.632
	Pore-water DON	0.617 *	0.128	−0.263	0.341	−0.117	−0.24
Oxygen	Surface sediment TOC	0.594 *	0.639 *	0.471	0.444	−0.756 **	−0.522
	Surface water DO	0.037	0.534 *	0.651 *	0.052	−0.369	−0.103
	Bottom DO	0.066	0.311	0.591 *	0.243	−0.074	0.238

* *p* < 0.05, ** *p* < 0.01.

## Data Availability

Data are available from authors upon request.

## Supplementary Materials

The following supporting information can be downloaded at: https://www.mdpi.com/article/10.3390/ijerph19094958/s1, Text S1: HIX value of water-extractable organic matter (WEOM) in sediment; Figure S1: Schematic illustration of the incubation experiment between sample vials (sediment and bottom water) and control vials (bottom water); Figure S2: Schematic illustration of the WEOM experiment; Figure S3: Changes in the optical properties of DOM and WEOM from the sediment of three weirs (Gangcheon, Yeoju, Ipo weirs) during the incubation period) [25,63,64,65,66].
