# Supplementary material for: Changes in the Dissolved Organic Matter Characteristics Released from Sediment According to Precipitation in the Namhan River with Weirs: A Laboratory Experiment
Source: Int J Environ Res Public Health. 2022 Apr 19;19(9):4958. doi: 10.3390/ijerph19094958 (PMC9101428; doi:10.3390/ijerph19094958)
Supplement: Supplementary file 1 [file ijerph-19-04958-s001.zip › ijerph-1650043-supplementary.pdf]

Supplementary Materials

Changes in the Dissolved Organic Matter Characteristics Released from Sediment According to Precipitation in the Namhan River with Weirs: A Laboratory Experiment

Haeseong Oh and Jung-Hyun Choi *

Department of Environmental Science and Engineering, Ewha Womans University, 52, Ewhayeodae-gil, Seodaemun-gu, Seoul 03760, Korea; hs1226@ewhain.net
* Correspondence: jchoi@ewha.ac.kr

Text S1: HIX value of water-extractable organic matter (WEOM) in sediment

Water-extractable organic matter (WEOM) was measured in sediments collected at 2 cm depth at Gangcheon, Yeoju and Ipo weir in August 2016 and May 2017. A 2 g subsample of each surface sediment sample (0–2 cm) was shaken (150 rpm) in 20 ml of deionized water for 1 hr (Figure S2) [1–3]. The sample was centrifuged (10,000 rpm, 10 min), and the supernatant was passed through a 0.7 μm glass filter (Whatman GF/F). The filtered supernatant was incubated to examine the microbial activity of organic matter from sediment. Incubation was conducted in a dark chamber where the ambient temperature was kept constant at 20 °C for up to 7 days. A total of 6 samples were incubated at each site. At 0, 3 and 7 days of incubation, 2 samples from each site were sacrificed for analysis. After incubation, samples filtered through a 0.7 μm glass filter (Whatman GF/F), and analyzed for HIX. The methods of HIX measurement are the same as the HIX method in manuscript.

The results of WEOM HIX value is shown in Figure S3. The WEOM HIX values of initial samples were in the range of 0.29–0.66. Since the WEOM HIX value is less than 10, samples was not humified and contained more oxygen-containing functional groups [4]. WEOM HIX that remained low and only showed a slight increase during the incubation period (Day 3 and 7) is ranged 0.29–1.33.There were no significantly difference in WEOM HIX values between the initial and incubated samples(one-way ANOVA, $p>0.05$). It means that the organic matter released from the sediment to the water layer is not humified by microorganism in the water layer during incubation period [5].

References

1. Hishi, T.; Hirobe, M.; Tateno, R.; Takeda, H. Spatial and temporal patterns of water-extractable organic carbon (WEOC) of surface mineral soil in a cool temperate forest ecosystem. *Soil Biol. Biochem.* **2004**, *36*, 1731–1737. https://doi.org/10.1016/j.soilbio.2004.04.030.
2. Li, Z.; Shengrui, W.; Haichao, Z.; Yanping, L.; Shouliang, H.; Weibin, Q.; Jie, C. Using multiple combined analytical techniques to characterize water extractable organic nitrogen from Lake Erhai sediment. *Sci. Total Environ.* **2016**, *542*, 344–353. https://doi.org/10.1016/j.scitotenv.2015.10.039.
3. Zhang, P.; Cao, C.; Wang, Y.H.; Yu, K.; Liu, C.; He, C.; Wang, J.J. Chemodiversity of water-extractable organic matter in sediment columns of a polluted urban river in South China. *Sci. Total Environ.* **2021**, *777*, 146127. https://doi.org/10.1016/j.scitotenv.2021.146127.
4. McKnight, D.M.; Boyer, E.W.; Westerhoff, P.K.; Doran, P.T.; Kulbe, T.; Andersen, D.T. Spectrofluorometric characterization of dissolved organic matter for indication of precursor organic material and aromaticity. *Limnol. Oceanogr.* **2001**, *46*, 38–48. https://doi.org/10.4319/lo.2001.46.1.0038.
5. Hansen, A.M.; Kraus, T.E.; Pellerin, B.A.; Fleck, J.A.; Downing, B.D.; Bergamaschi, B.A. Optical properties of dissolved organic matter (DOM): Effects of biological and photolytic degradation. *Limnol. Oceanogr.* **2016**, *61*, 1015–1032. https://doi.org/10.1002/lno.10270.

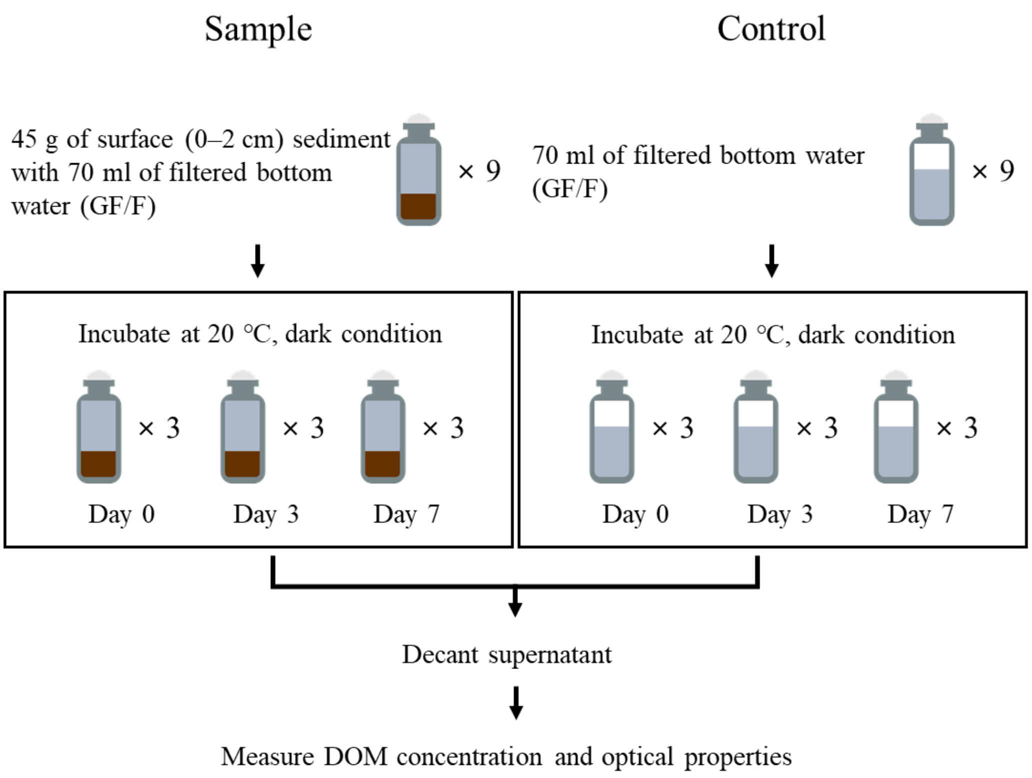

Figure S1. Schematic illustration of the incubation experiment between sample vials (sediment and bottom water) and control vials (bottom water).

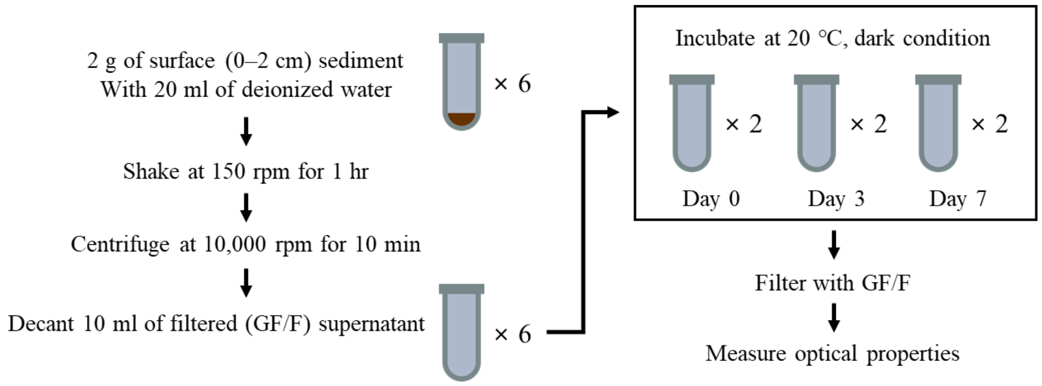

Figure S2. Schematic illustration of the WEOM experiment.

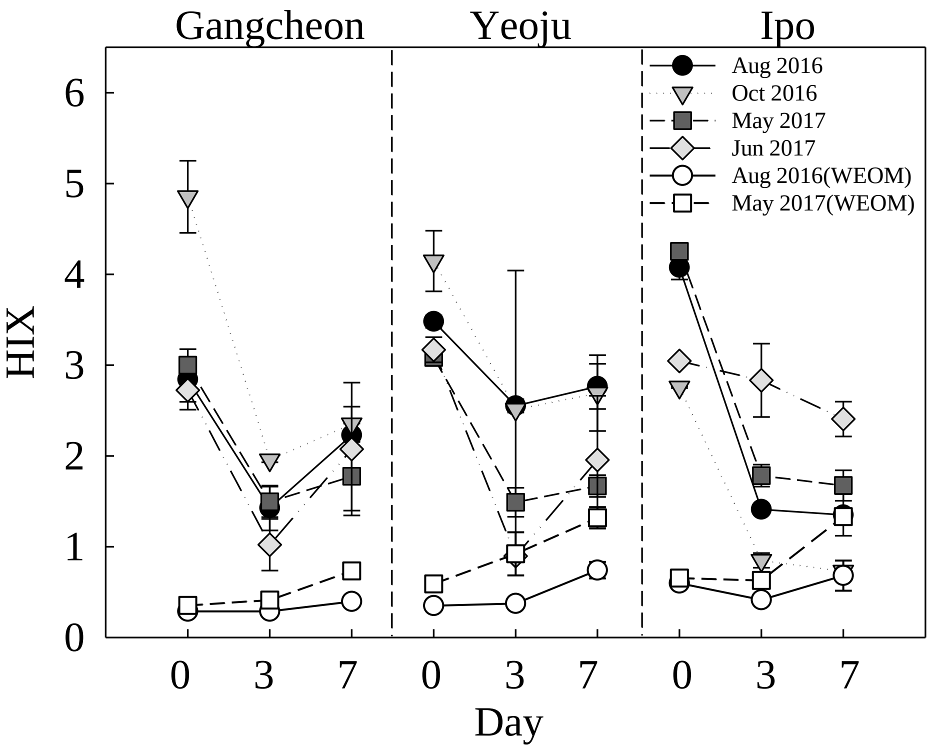

Figure S3. Changes in the optical properties of DOM and WEOM from sediment of three weirs (Gangcheon, Yeoju, Ipo weir) during incubation period).
